# Bacteriophage T4 Escapes CRISPR Attack by Minihomology Recombination and Repair

**DOI:** 10.1128/mBio.01361-21

**Published:** 2021-06-22

**Authors:** Xiaorong Wu, Jingen Zhu, Pan Tao, Venigalla B. Rao

**Affiliations:** a Bacteriophage Medical Research Center, Department of Biology, The Catholic University of America, Washington, DC, USA; University of Pittsburgh

**Keywords:** bacteriophage T4, CRISPR-Cas genome editing, end joining, phage counterdefense, UvsX recombinase, homologous recombination

## Abstract

Bacteria and bacteriophages (phages) have evolved potent defense and counterdefense mechanisms that allowed their survival and greatest abundance on Earth. CRISPR (clustered regularly interspaced short palindromic repeat)-Cas (CRISPR-associated) is a bacterial defense system that inactivates the invading phage genome by introducing double-strand breaks at targeted sequences. While the mechanisms of CRISPR defense have been extensively investigated, the counterdefense mechanisms employed by phages are poorly understood. Here, we report a novel counterdefense mechanism by which phage T4 restores the genomes broken by CRISPR cleavages. Catalyzed by the phage-encoded recombinase UvsX, this mechanism pairs very short stretches of sequence identity (minihomology sites), as few as 3 or 4 nucleotides in the flanking regions of the cleaved site, allowing replication, repair, and stitching of genomic fragments. Consequently, a series of deletions are created at the targeted site, making the progeny genomes completely resistant to CRISPR attack. Our results demonstrate that this is a general mechanism operating against both type II (Cas9) and type V (Cas12a) CRISPR-Cas systems. These studies uncovered a new type of counterdefense mechanism evolved by T4 phage where subtle functional tuning of preexisting DNA metabolism leads to profound impact on phage survival.

## INTRODUCTION

Bacteriophages and bacteria are the most abundant and widely distributed organisms on Earth. They have been at “war” with each other, for millions of years, which led to the evolution of defense and counterdefense systems and their greatest diversity and ecological balance in our biosphere. Two of the well-characterized bacterial defense systems are restriction-modification enzymes (1, 2) and CRISPR (clustered regularly interspaced short palindromic repeat)-Cas (CRISPR-associated) genome-cleaving complexes (3–5). The counterdefense mechanisms that phages evolved are not as well understood. Genome modifications such as glucosylation and cytosine hydroxymethylation (ghmC) (6–9), anti-restriction (10), and anti-CRISPR (11–16) proteins constitute some of the counterdefense mechanisms.

**FIG 1:**
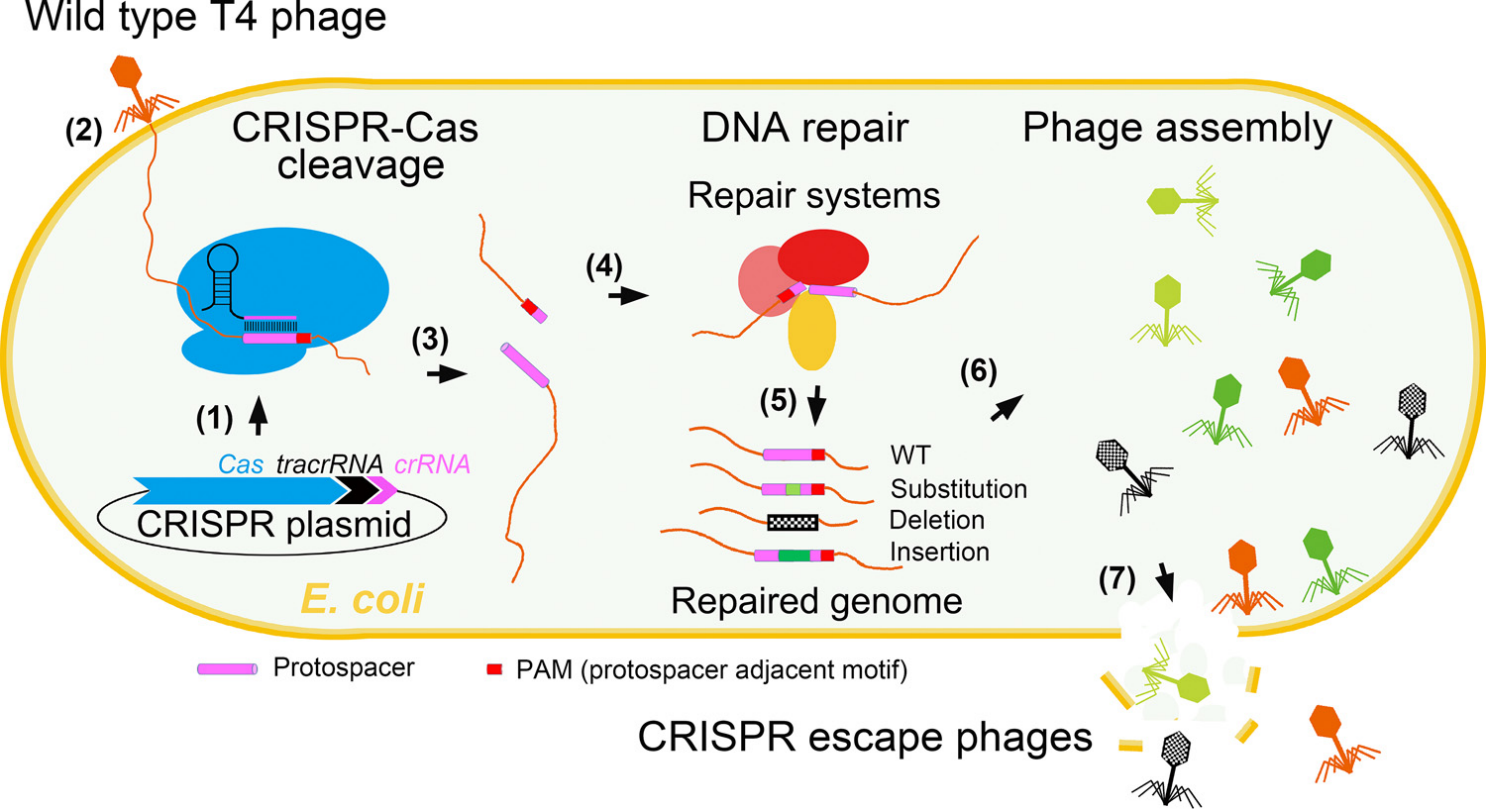
Schematic of phage T4 escape under CRISPR pressure. The CRISPR-Cas plasmid that constitutively expresses Cas9 (or Cas12a), *trans*-activating CRISPR RNA (tracrRNA), and crRNA was introduced into E. coli. The expressed components form CRISPR-Cas genome editing “effector complexes” (step 1). After wild-type (WT) T4 phage infection (step 2), the effector complex recognizes protospacer sequence in the T4 genome and makes a double-strand break (step 3). Joining of broken ends with the aid of repair systems (step 4) leads to the generation of escape mutations, including deletions, substitutions, or insertions (step 5). The restored T4 genome that is resistant to CRISPR will be packaged, and infectious viral particles will be assembled (step 6). Lysis of E. coli envelope leads to release of CRISPR escape mutant phages (step 7).

CRISPR-Cas is an adaptive immune system of bacteria that produces a series of RNAs (CRISPR RNAs [crRNAs]) from arrays of “spacers,” ∼20-nucleotide (nt) phage genome sequences adjacent to a 3- to 6-nt sequence called PAM (protospacer adjacent motif), that bacteria acquire during previous exposure to phages (17–19). These crRNAs form “effector complexes” with Cas nucleases and surveil the intracellular space of bacteria. When invaded by a phage, the effector complex recognizes the identical “protospacer” sequence present in the phage genome next to a PAM sequence and introduces a double-strand break (17, 18, 20, 21) (Fig. 1). The double-strand breaks are lethal because, unless restored by end joining, the broken genome sections will be degraded by nucleases and no phage progeny will be produced (22).

Recently, we discovered that the ghmC-modified T4 genome provides substantial counterdefense, by resisting cleavage by type II Cas9 and type V Cas12a effector complexes (6, 9). Furthermore, this resistance leads to unexpectedly high frequency of CRISPR escape, often by the time a plaque is formed from a single phage infection (23); however, the mechanisms were unknown. Moreover, since the CRISPR-Cas was targeted to essential structural genes (portal and major capsid protein genes), escape was necessarily biased toward the selection of silent or missense mutations that can restore gene function (23).

Here, by systematically targeting CRISPR-Cas to various nonessential regions of T4 genome, we asked, what is the predominant mechanism by which phage T4 defends itself against CRISPR attack? We found, surprisingly, that deletions are created at the cleavage site, from as short as 17 bp to as large as 18 kbp. Close examination of the deletions revealed that they all contained a short sequence repeated on either side of the deleted sequence. Such deletions were observed at every location targeted on the T4 genetic map and in response to both type II (Cas9) or type V (Cas12a) CRISPR attacks. Analysis of numerous deletions suggests an unusual recombination mechanism mediated by phage T4-encoded recombinase UvsX, in which the broken genomic fragments are paired through short stretches of sequence identity, as few as 3 or 4 nucleotides (minihomology sites), that then leads to replication, repair, and stitching of the paired regions. These results highlight how phages evolved powerful counterdefense mechanisms against CRISPR attack by functional tuning of the existing DNA metabolism enzymes, imparting broad selective advantages for their survival.

## RESULTS

### Evolutionary signatures of phage T4 CRISPR escape mutants.

**FIG 2:**
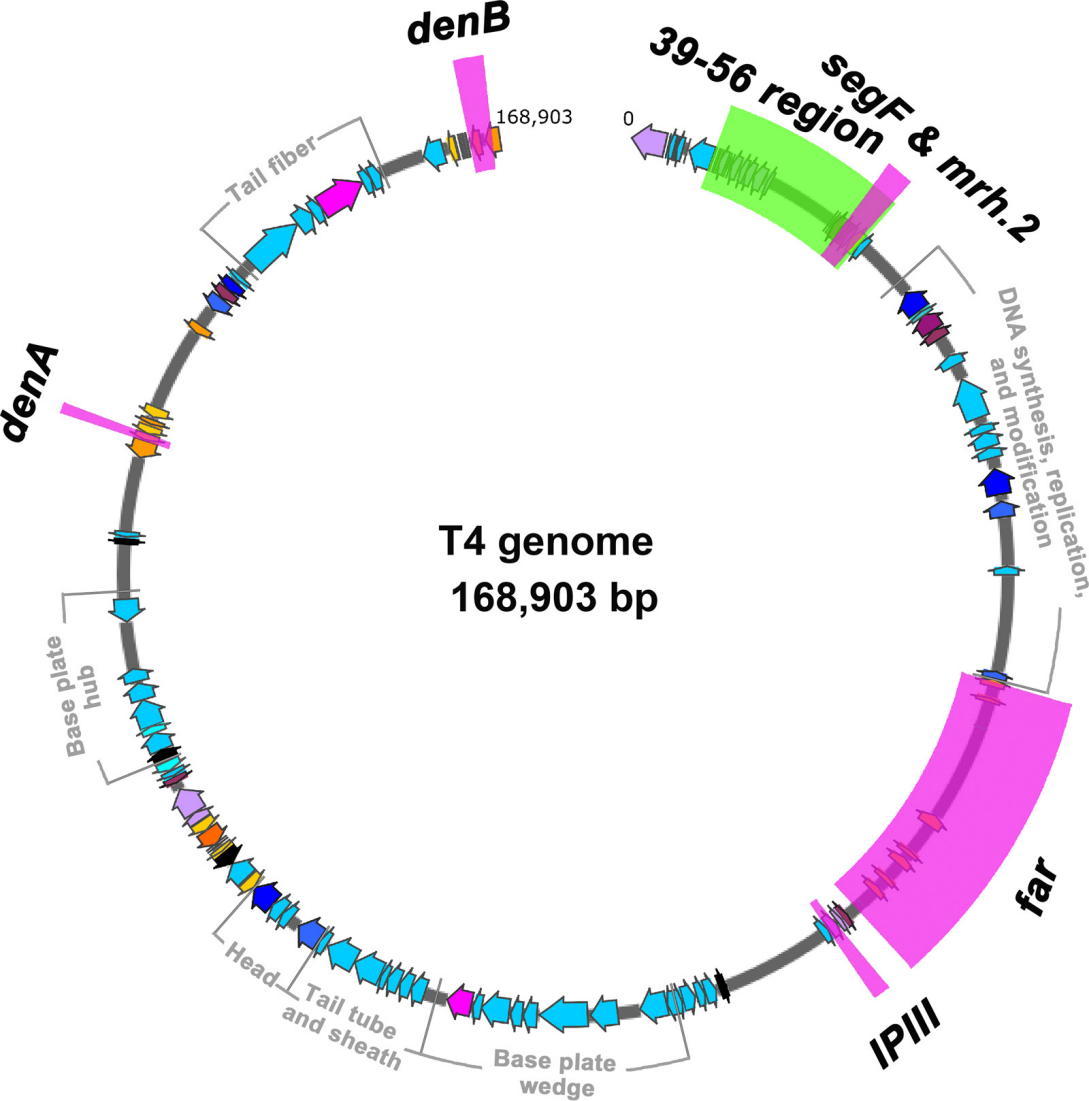
Schematic showing CRISPR-Cas targets on T4 genome. The 168,903-bp T4 phage genome is represented by the open circle (dark gray). The genes are shown by arrows on the circle in different transcription directions and colors. Essential gene clusters for viable phage reproduction are labeled and indicated in light gray lines outside the circle. The CRISPR-Cas targets designed in this work, i.e., *denA*, *denB*, *segF*, *mrh.2*, *IPIII*, and the *far* region, are shown in magenta boxes. The *39-56* region, the nonessential deletion reported by Homyk and Weil (70), is shown in a green box.

**FIG 3:**
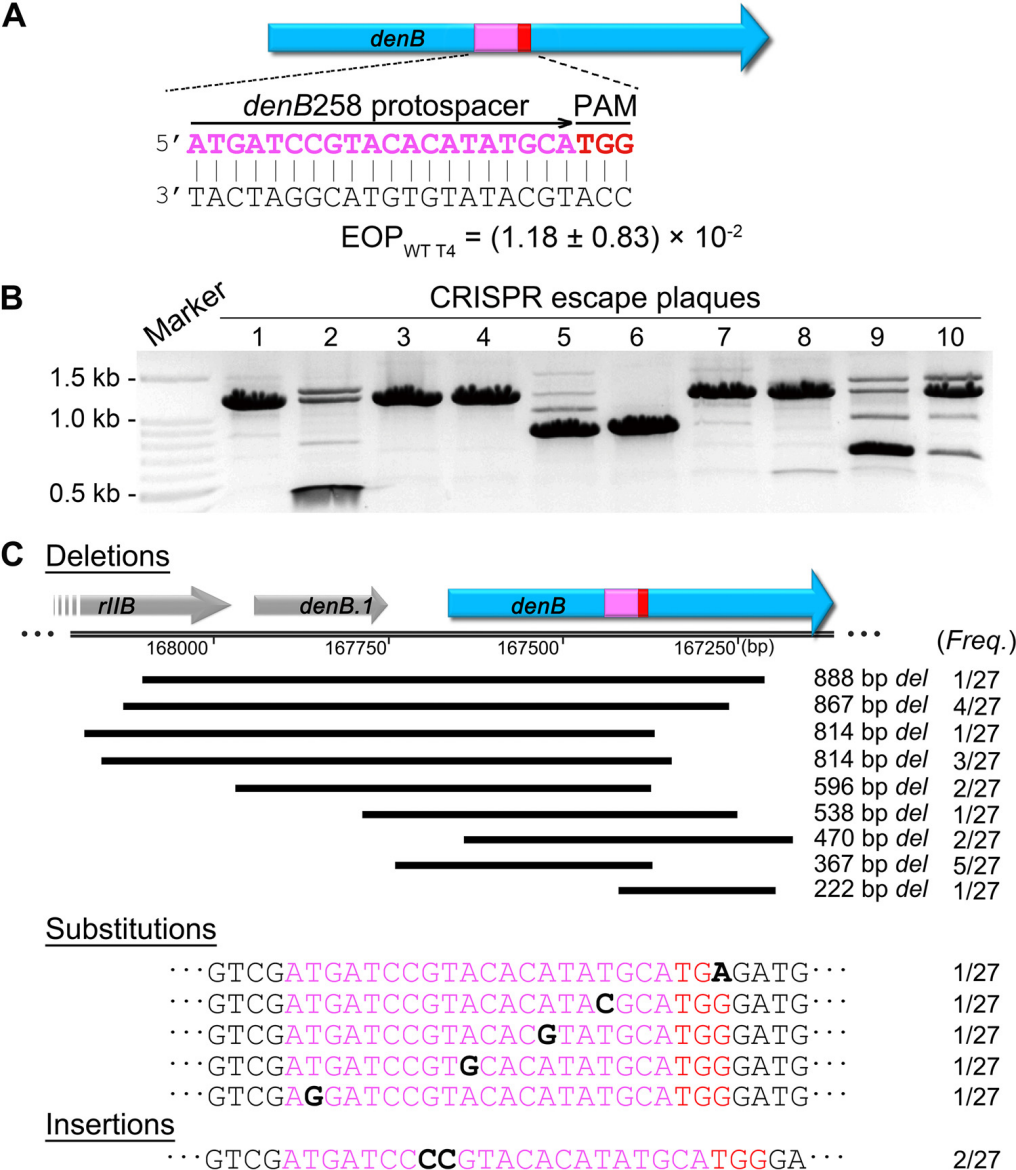
Phage T4 plaques produced under CRISPR pressure represent the evolutionary signatures of escape mutants. (A) Schematic showing the *denB*258 protospacer (magenta) location on the *denB* gene (blue rectangular box with an arrow in the direction of transcription) and PAM (red) sequences. The EOP was determined by dividing the number of plaques produced by WT phage infection of *denB*258 CRISPR E. coli by the number of plaques produced by infection of control E. coli lacking the *denB*258 CRISPR plasmid. (B) Evolutionary signature of individual G1 CRISPR escape plaques were determined by PCR. The amplified DNAs were electrophoresed on an agarose gel (lanes 1 to 10). The “Marker” lane contained standards ranging in size from 100 bp to 3,000 bp. The sizes of main bands from lane 1 to 10 were 1,286, 398, 1,286, 1,286, 919, 919, 1,286, 1,286, 691, and 1,286 bp, respectively. The 12,86-bp bands indicate PCR products from WT or substitution mutant phage, while other shorter bands indicate PCR products from deletion mutant phages. (C) Individual bands from the agarose gel were isolated, and the DNA was extracted and sequenced. The sequences of all the escape mutations, 27 in total, are shown (WT sequences are not shown). Deletions with different lengths and locations in the genome are shown in black bars (the scale corresponds to the T4 genome [24]). Substitutions and insertions in the protospacer or PAM are shown in black bold font. The numbers on the side show the frequency (*Freq*.) of each mutation.

In order to minimize bias and capture all types of CRISPR escape mutants (Fig. 1), we chose a nonessential T4 gene *denB* as a target (Fig. 2). DenB is an endonuclease that degrades cytosine DNA of the Escherichia coli genome within minutes after phage infection. The ghmC-modified phage genome however is resistant to DenB cleavage (24, 25). The spacer RNA was designed such that the CRISPR-Cas9 effector complex introduces a double-strand break in the central region of the *denB* gene, which results in inactivation of DenB function as well as loss of genome integrity (Fig. 1 and 3A). Viable phage could be produced upon phage infection only if the broken ends were rejoined by repair mechanisms that would also introduce mutations making the protospacer region refractory to CRISPR-Cas9 cleavage (Fig. 1).

The plating efficiency of the wild-type (WT) T4 phage (efficiency of plating WT T4 phage [EOP_WT T4_]) on the *denB* spacer RNA-expressing CRISPR E. coli was on the order of ∼10^−2^, indicating a high frequency of escape. The *denB* gene from these plaques was PCR amplified and analyzed by agarose gel electrophoresis. Remarkably, each plaque showed a series of bands and a distinct band pattern (Fig. 3B), presumably representing the products of repair mechanisms employed by phage T4 to join the double-strand breaks. When the band corresponding to the WT position was sequenced, we found, similar to our previous studies, some WT sequences, single-nucleotide substitution mutations, and two-nucleotide (CC) insertions in the protospacer sequence among these first generation (G1) plaques (Fig. 3C). The WT sequences represent some uncleaved genomes that are expected to be present in each “escaped” G1 plaque. This fraction diminished progressively when these plaques were replica plated in subsequent G2 and G3 generations (23; data not shown). Unexpectedly, however, a series of short DNA bands were present virtually in every plaque (Fig. 3B), which we have not observed when essential genes were targeted (23).

When these DNA bands were purified and sequenced, we found a series of deletions in the *denB* gene in which the PAM and protospacer sequences were also deleted (Fig. 3C). Another unexpected observation was that identical deletion endpoints were observed in independently isolated plaques (e.g., the 367-bp deletion was recovered in five different plaques; Fig. 3C). These data demonstrated that a predominant mechanism that joins CRISPR-generated double-strand breaks in T4 phage genome, at least in the *denB* gene, was through deletions (Fig. 3C). Though the size of deletions varied, the process was not random because, otherwise, we would not have found deletions with precise endpoints in independent isolates.

Multiple deletion bands were found in each plaque, and at different intensities, which probably reflect the time at which that particular deletion arose and its survival fitness. Each plaque originates from a single phage infection of a single bacterium. A deletion mutant arising early and/or more resistant to Cas9 cleavage would accumulate more phage progeny in that plaque, and hence generates a higher intensity of that deletion band compared to those that arose later or are less fit. Each plaque, thus, represented the evolutionary signature of CRISPR escape.

### Deletions represent a predominant mechanism used by T4 phage to join type II Cas9-cleaved genomes.

**FIG 4:**
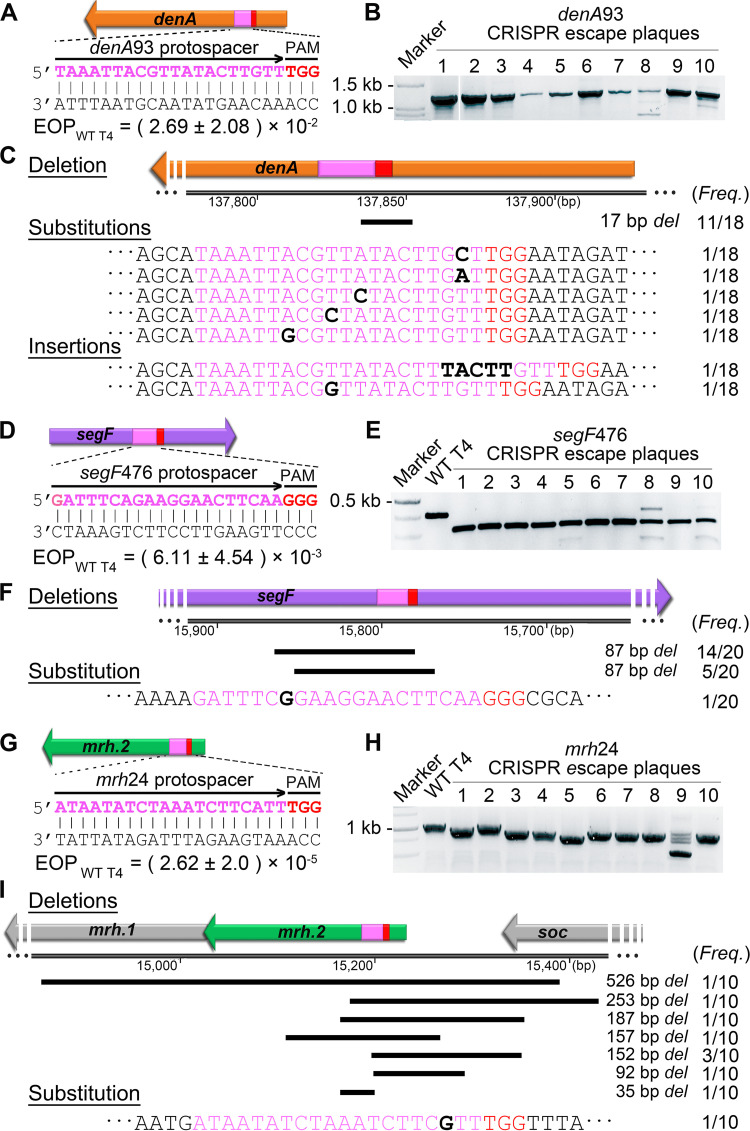
Deletions represent a common end-joining mechanism in CRISPR escape T4 phages. The T4 genes targeted by CRISPR-Cas9 are shown in different colors: *denA* (A), *segF* (D), and *mrh*.*2* (G). For each targeted gene, the protospacer (magenta) and PAM (red) sequences are shown. The EOP was determined by dividing the number of plaques produced by WT phage infection of CRISPR E. coli by the number of plaques produced by infecting control E. coli lacking the CRISPR plasmid. Twenty-six G1 plaques from *denA*93 infection, 20 from *segF*476 infection, and 9 from *mrh*24 infection were picked, and the respective gene was amplified by PCR and sequenced. (B, E, and H) Agarose gels showing the amplified DNA from CRISPR escape plaques from the respective infection. The “Marker” lane contained standards ranging in size from 100 bp to 3,000 bp. For the *denA*93 spacer, the sizes of the main bands from lane 1 to 10 were 1,177 (WT T4 phage), 1,160 (deletion mutant phage), 1,160, 1,177, 1,177, 1,160, 1,177, 1,177, 1,160, and 1,160 bp, respectively (B). For the *segF*476 spacer, the size of the band from WT T4 is 410 bp, while the main bands from lane 1 to 10 are 323 bp (E). For the *mrh*24 spacer, the “Marker” lane contained standards ranging in size from 250 bp to 25 kbp, and the size of band from WT T4 is 1,061 bp, while the main bands from lanes 1 to 10 are 969, 1,061, 909, 904, 808, 909, 909, 874, 535, and 909 bp (H). (C, F, and I) Sequencing results of G1 CRISPR escape plaques. (C) Out of 26 *denA*93 plaques, 18 were mutants. Eleven show an identical 17-bp deletion (black bar), five show substitution in protospacer, and two show TACTT or G insertion. (F) Nineteen out of 20 plaques from *segF*476 show two types of 87-bp deletions, and one shows a substitution in protospacer. (I) Out of 10 plaques from *mrh*24, one is an A-G point mutation, while the others have deletions of various lengths. The numbers on the side show the frequency (*Freq*.) of each mutation.

To determine whether the high frequency of deletions observed in *denB* CRISPR escape mutants is a common mechanism, the same type of analysis was performed with three additional nonessential genes; *denA*, *segF*, and *mrh.2*, from different locations of the T4 genome (Fig. 2) (24). The EOP_WT T4_ on CRISPR E. coli expressing the respective spacers were on the order of 10^−2^, 10^−3^, and 10^−5^, respectively (Fig. 4). DNAs from at least 10 plaques for each gene were amplified and analyzed by agarose gel electrophoresis and sequencing. The data show that deletions were found among the CRISPR escape mutants of all three genes. Remarkably, in the case of *denA* (Fig. 4A to C), the same 17-bp deletion was repeatedly observed, and in the case of *segF*, an 87-bp deletion was repeatedly found, although fainter bands representing longer deletions were also observed in some of the plaques (Fig. 4D to F). On the other hand, in the case of the *mrh.2* gene, a variety of deletions were observed corresponding to 35, 92, 152, 157, 187, 253, and 526 bp (Fig. 4G to I).

These data sets further demonstrated that the length of the deletion varied in different genes, and also within the same gene, and some of the same deletions repeatedly occurred in phages evolving independently, as was observed in the case of the *denB* gene. Moreover, deletion is again found to be a dominant mechanism used by phage T4 to repair and rejoin Cas9-cleaved ends, although (mostly single) substitution mutants were also found but less commonly when these nonessential genes were targeted.

### Deletions occur through a minihomology-mediated end-joining mechanism.

**FIG 5:**
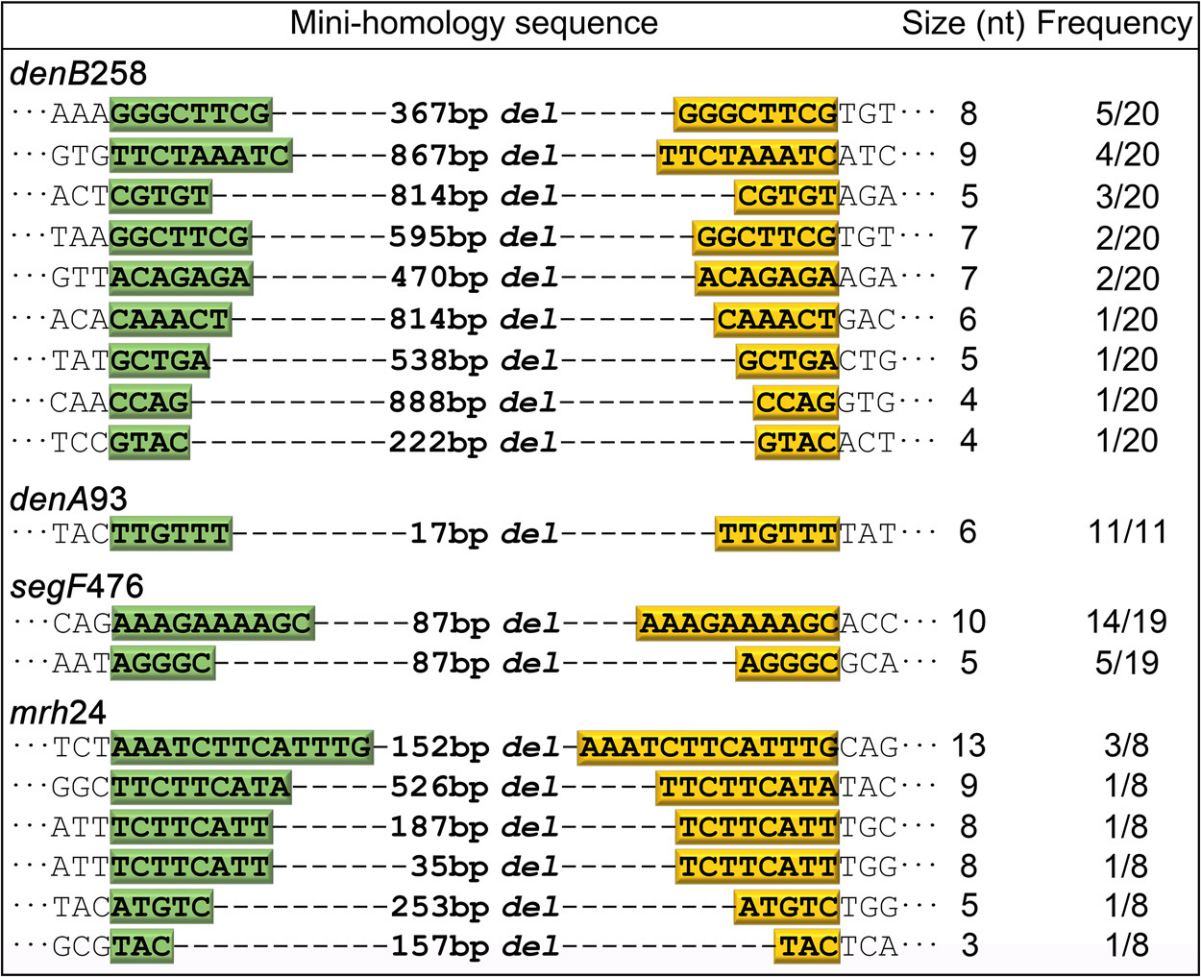
Deletions occur through a minihomology-mediated end-joining mechanism. Each CRISPR escape phage containing a deletion that arose from targeting four different nonessential genes from different locations of the T4 genome showed short stretches of sequence identity (minihomology sequences) flanking the deletion endpoints. The minihomology sequences on the left are highlighted in green, and the ones on the right are highlighted in yellow. The size of each minihomology is shown in the middle column. The frequency of each deletion out of the number of total deletions from each gene is shown in the rightmost column.

It was apparent from the above data that the deletion end-joining mechanism we have discovered must involve certain “rules” because the deletion endpoints were precise in independently evolved phage mutants. Of ∼100 CRISPR escape plaques sequenced, 58 were deletions, out of which 18 were unique deletions and the rest were repeats of these deletions in independent isolates (Fig. 5). Closer examination of these deletions revealed that each deletion was flanked by a short stretch of identical sequence, ∼3 to 13 nucleotides long, in the parental genome. We refer to these repeat sequences as “minihomology” sites. In the deleted sequence, only one of the two minihomology repeats was retained, which means that the other repeat was lost as a result of recombination. Since the deletion occurred precisely at these sites in independent recombination events and in all four genes tested, it can be concluded that this end-joining mechanism recognizes very short stretches of complementarity, not commonly observed in classic homologous recombination mechanisms, which typically require a minimum of ∼50- to 150-nt sequence identity (26). Otherwise, it would lead to genome instability if minihomology recombination occurred at any significant frequency.

### The minihomology recombination mechanism also joins type V Cas12a-cleaved genomes.

**FIG 6:**
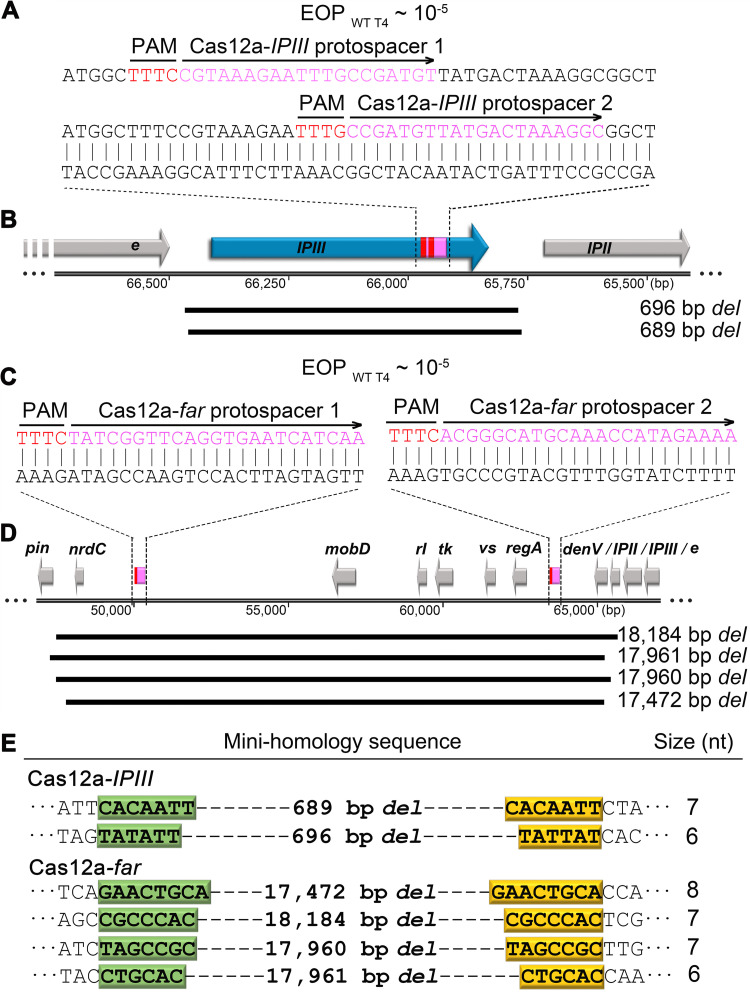
The minihomology recombination mechanism also joins type V Cas12a-cleaved genomes. The protospacers (magenta) and PAM (red) sequences of CRISPR-Cas12a, which target *IPIII* (A) and the *far* region (C), are shown. The EOP was determined by dividing the number of plaques produced by WT phage infection of CRISPR E. coli by the number of plaques produced by infecting control E. coli lacking the CRISPR plasmid. G1 plaques from Cas12a*-IPIII* and Cas12a*-far* infection were picked, and the genetic signature of each plaque was analyzed by PCR and sequencing. The lengths and locations of deletions from Cas12a*-IPIII* (B) and Cas12a*-far* (D) on the T4 genome are shown in black bars. (E) The left column shows the minihomology sequences flanking each deletion endpoints, the left ones are highlighted in green, and the ones on the right in yellow. The size (number of nucleotides) of each minihomology site is shown in the right column.

Next, we asked whether this minihomology recombination mechanism also operates on type V Cas12a-cleaved genomes. We chose two additional nonessential regions of T4 genome, the *IPII*/*IPIII* region and the *far* (folate analog resistance) region (Fig. 2) to address this question. Cas12a is known to cleave the ghmC-modified phage T4 genome more efficiently than Cas9 (6). To impose even stricter conditions for end joining, two spacers with overlapping protospacer targets were used (Fig. 6A). Having two overlapping spacers would further increase the frequency of target cleavage at the target site, requiring efficient end joining to offset the degradation of cleaved genomic fragments. Not unexpectedly, the EOP_WT T4_ under double spacer pressure was quite low, on the order of ∼10^−5^. Nevertheless, when sequenced, virtually every plaque produced from this infection carried a deletion at the target site. Shown in the figure are the most frequent 696- and 689-bp deletions that used 6- and 7-nt minihomology sites, TATATT and CACAATT, respectively, flanking the deleted sequence (Fig. 6B and E).

To further increase the stringency, we targeted two protospacers separated by ∼15 kbp in the *far* region (Fig. 2 and 6C). Hence, in this case, the T4 genome will be cleaved by Cas12a at two target sites that are ∼15 kbp apart. Previous genetic studies (27) identified an ∼13-kbp deletion spanning this region, suggesting that this entire fragment is nonessential and could be deleted. Remarkably, when we examined the CRISPR escaped plaques from this infection, virtually every plaque showed a large ∼17- to 18-kbp deletion. Each deletion once again was marked by a 6- to 8-nt minihomology site flanking the deleted sequence (Fig. 6D and E). This demonstrated that, even when multiple cleavages occurred on the genome and even when the cleaved fragments were separated by a large intervening segment, the genomic pieces can be brought together and covalently rejoined, attesting to the robustness of the minihomology recombination/repair mechanism.

### Features of the minihomology-mediated end-joining mechanism.

Further analysis of the minihomology sequences showed no sequence specificity or a common feature that correlated with recombination at these sites. Therefore, it is unclear why certain minihomology sequences were frequently used whereas others were used infrequently or not at all. For instance, of the 15 possible 7-nt minihomology sequences flanking the *denB*258 spacer, only two were used, and of the 33 6-nt minihomology sequences, only one was used (see Fig. S1 in the supplemental material). However, some trends were apparent. First, longer minihomology sequences were more frequently used than the shorter ones. The frequencies of usage for longer sequences were 19%, 100%, 75%, and 37.5% for *denB*258 (Fig. S1), *denA*93 (Fig. S2), *segF*476 (Fig. S3), and *mrh*24 (Fig. S4) spacers, respectively, compared to 4.8%, 0%, 25%, and 12.5% for shorter homologies. Second, sequences with higher GC content were more frequently used than the ones with lower GC content in the studied *denB*258, *segF*476, and *mrh*24 spacers. A good example of this is the *segF*476 CRISPR escape mutants which show that the 5-nt AGGGC sequence (80% GC) was picked 25% of the time, whereas 14 6- or 7-nt sequences or 25 other 5-nt sequences with lower GC content were not picked at all. This may be because longer or higher GC sequences more efficiently pair and generate more stable recombination intermediates leading to successful end joining (Fig. S3). Third, the minihomology sequences closer to the Cas9 cleavage site appear to be more frequently used than the distant ones. An extreme example was found in the case of *denA*93 where 100% of the deletions (11 out of 11) occurred at the two sites that are closest to the Cas9 cleavage site (Fig. S2). Finally, one of the two ends of the deletion, particularly the end on the 5′ side of the PAM sequence, is often much closer to the double-strand break compared to the other end. For example, in the three most frequent deletions using the *denB*258 spacer, the deletion on the 5′ side of the PAM site was at 30, 143, and 35 bp from the Cas9 break compared to 337, 724, and 778 bp, respectively, on the opposite side (Fig. S1). This means that the end containing the PAM sequence was less susceptible to nuclease digestion following Cas9 cleavage.

10.1128/mBio.01361-21.1 FIG S1 Minihomology sequences flanking the *denB*258 protospacer. Shown are the possible minihomology sequences, short stretches of identical sequences ranging from 4 to 9 nucleotides (leftmost column), their GC content (percent), and frequency (percent) of their usage among the CRISPR escape plaques. The minihomology sequences were detected using the computing engine FAIR (Finding All Internal Repeats). A ∼900-bp genomic sequence on the non-PAM side and ∼300 bp on the PAM side flanking the *denB*258 CRISPR cleavage site (vertical line) was used as an input sequence. Each minihomology sequence (the second column from left [only some of the sequences in each length range are shown due to space restrictions]) is shown as a small vertical bar on the horizontal line representing the genome sequence, and those sequences detected in sequenced CRISPR escape plaques are shown in bold type. The protospacer sequence is shown as a magenta arrow, and the PAM site is shown as a red box. Download FIG S1, TIF file, 2 MB. Copyright © 2021 Wu et al. 2021 Wu et al. https://creativecommons.org/licenses/by/4.0/ This content is distributed under the terms of the Creative Commons Attribution 4.0 International license.

10.1128/mBio.01361-21.2 FIG S2 Minihomology sequences flanking the *denA*93 protospacer. Shown are the possible minihomology sequences, short stretches of identical sequences ranging from 5 to 8 nucleotides (leftmost column), their GC content (percent), and frequency (percent) of their usage among the CRISPR escape plaques. The minihomology sequences were detected using the computing engine FAIR. A ∼100-bp genomic sequence on either side flanking the *denA*93 CRISPR cleavage site (vertical line) was used as an input sequence. Each minihomology sequence (second column from left) is shown as a small vertical bar on the horizontal line representing genome sequence, and those sequences detected in sequenced CRISPR escape plaques are shown in bold type. The protospacer sequence is shown as a magenta arrow, and the PAM site is shown as a red box. Download FIG S2, TIF file, 0.5 MB. Copyright © 2021 Wu et al. 2021 Wu et al. https://creativecommons.org/licenses/by/4.0/ This content is distributed under the terms of the Creative Commons Attribution 4.0 International license.

10.1128/mBio.01361-21.3 FIG S3 Minihomology sequences flanking the *segF*476 protospacer. Shown are the possible minihomology sequences, short stretches of identical sequences ranging from 5 to 10 nucleotides (leftmost column), their GC content (percent), and frequency (percent) of their usage among the CRISPR escape plaques. The minihomology sequences were detected using the computing engine FAIR. A ∼200-bp genomic sequence on either side flanking the *segF*476 CRISPR cleavage site (vertical line) was used as an input sequence. Each minihomology sequence (the second column from left [only some of the sequences in each length range are shown due to space restrictions]) is shown as a small vertical bar on the horizontal line representing genome sequence, and those sequences detected in sequenced CRISPR escape plaques are shown in bold type. The protospacer sequence is shown as a magenta arrow, and the PAM site is shown as a red box. Download FIG S3, TIF file, 1.6 MB. Copyright © 2021 Wu et al. 2021 Wu et al. https://creativecommons.org/licenses/by/4.0/ This content is distributed under the terms of the Creative Commons Attribution 4.0 International license.

10.1128/mBio.01361-21.4 FIG S4 Minihomology sequences flanking the *mrh*24 protospacer. Shown are the possible minihomology sequences, short stretches of identical sequences ranging from 3 to 13 nucleotides (leftmost column), their GC content (percent), and frequency (percent) of their usage among the CRISPR escape plaques. The minihomology sequences were detected using the computing engine FAIR (Finding All Internal Repeats). A ∼300-bp genomic sequence on either side flanking the *mrh*24 CRISPR cleavage site (vertical line) was used as an input sequence. Each minihomology sequence (the second column from left [only some of the sequences in each length range are shown due to space restriction]) is shown as a small vertical bar on the horizontal line representing genome sequence, and those sequences detected in sequenced CRISPR escape plaques are shown in bold type. The protospacer sequence is shown as a magenta arrow, and the PAM site is shown as a red box. Download FIG S4, TIF file, 2 MB. Copyright © 2021 Wu et al. 2021 Wu et al. https://creativecommons.org/licenses/by/4.0/ This content is distributed under the terms of the Creative Commons Attribution 4.0 International license.

### The phage T4 recombinase UvsX is essential for minihomology-mediated end-joining mechanism.

Recombinases that might be involved in the minihomology-mediated end-joining mechanism are the T4 homologous recombination proteins UvsX and UvsY and the E. coli recombinase RecA. UvsX is an ortholog of E. coli RecA and mammalian Rad52 (28–31). Although it is not an essential gene for phage viability (under laboratory conditions), UvsX is a key recombinase involved in all T4 recombination activities, including DNA strand exchange, recombination-directed replication (RDR), and homology-directed DNA repair (HDR) (32–35). UvsY, on the other hand, plays an accessory role and enhances the recombination efficiency of UvsX recombinase by stabilizing UvsX-ssDNA (single-stranded DNA) complexes (36–45). Similarly, the E. coli RecA binds to ssDNA to form a stable nucleoprotein filament that is important for paring to homologous sequence and directing DNA strand exchange (46–49).

**FIG 7:**
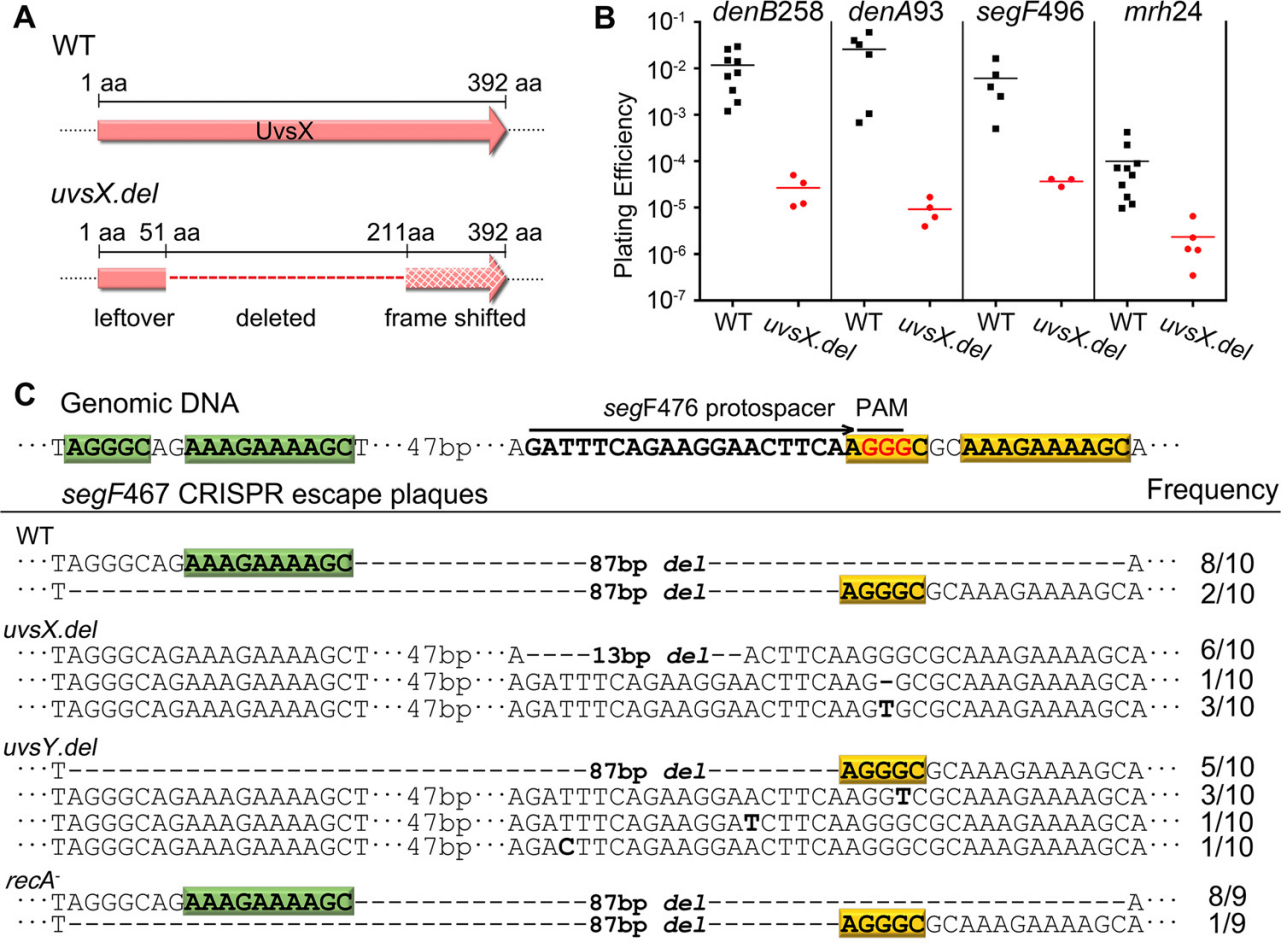
The phage T4 recombinase UvsX is essential for minihomology-mediated end-joining mechanism. (A) Schematic showing *uvsX.del* mutant construction. Of the 392-amino-acid (aa) WT UvsX protein, the sequence from aa 52 to aa 210 containing the ATPase and DNA binding domains was deleted by CRISPR editing (9). (B) EOP of WT and *uvsX.del* phages on E. coli DH5α as determined by plaque assay. (C) A total of 39 G1 CRISPR escape plaques produced from infection of *segF*476 CRISPR E. coli were sequenced. Ten plaques each were from WT phage, *uvsX.del* phage, or *uvsY.del* phage infections of *segF*476 CRISPR E. coli B834 (*recA*-plus), and nine plaques were from infection of *segF*476 CRISPR E. coli DH5α (*recA*-minus) with WT phage T4.

Since UvsX is nonessential (50), we first created a large deletion in the *uvsX* gene of the T4 genome by removing the coding sequence corresponding to its ATPase and DNA binding domains (51) using our CRISPR engineering strategy (9) (Fig. 7A). When this *uvsX.del* mutant phage was used for infection of CRISPR E. coli expressing various spacers as described above, the plating efficiency had dropped by up to 3 orders of magnitude compared to WT phage infection (Fig. 7B), suggesting that the *uvsX* gene is essential for CRISPR escape. Furthermore, when the CRISPR escape plaques of *segF* produced under the *uvsX.del* background were sequenced, they contained a single substitution mutation and 1- and 13-bp deletions involving no flanking minihomology sequences, unlike the minihomology-dependent 87-bp deletions found in the WT phage infection (Fig. 7C). These data demonstrate that the minihomology-mediated end joining was abolished in the absence of UvsX. The recovered CRISPR-resistant plaques (at ∼10^−5^ frequency; Fig. 7B) probably represent preexisting mutants present in the phage stocks, although they might also be derived from an alternative end-joining/repair mechanism (23).

We then tested the importance of the UvsY accessory protein by deleting most of the *uvsY* gene using our CRISPR engineering strategy. Infection of CRISPR E. coli with *uvsY.del* mutant phage also resulted in a reduction of plaque formation by ∼1 to 3 log units compared to WT phage infection. However, sequencing of the mutant plaques from these infections showed that about 30% of the plaques have the same 87-bp deletion as was found in the WT phage infection, whereas the rest were single-nucleotide substitutions in the protospacer sequence. These data further confirm that the UvsX recombinase is the key protein for minihomology-mediated end joining whereas UvsY merely reduces the efficiency of this mechanism, consistent with its well-documented accessory role (42).

Finally, we tested whether the E. coli RecA played a role in the minihomology-mediated end-joining mechanism by plating the WT phage on a *recA*-minus E. coli DH5α strain carrying the *segF*476 spacer and the CRISPR escape plaques were sequenced. Virtually every plaque from this set has shown the same 87-bp deletion, suggesting that RecA is not essential for the minihomology-mediated end-joining mechanism (Fig. 7C).

### A model for CRISPR-Cas end joining by minihomology recombination and repair.

**FIG 8:**
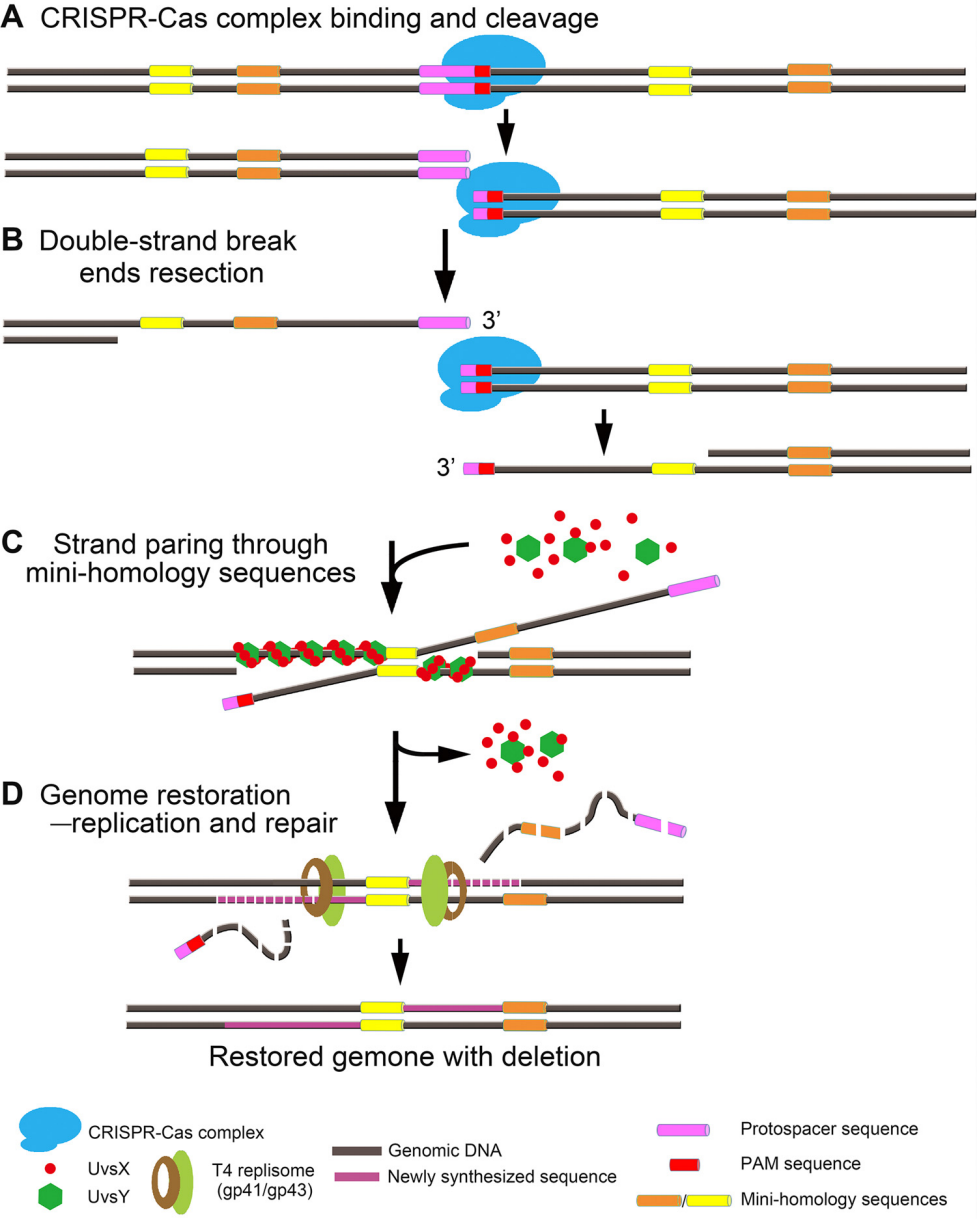
A model for minihomology-mediated end-joining mechanism. (A) Cas nuclease cleaves the target DNA. (B) Resection of Cas-cleaved ends by exonucleases generating 3′ overhangs. (C) UvsY and/or UvsX bind to 3′ overhang single-stranded DNA and promote strand exchange (pairing) and annealing through minihomology sequences. (D) Replication and repair proteins assemble. Genome integrity is restored by degradation of overhangs, extension of 3′ ends, and ligation of nicks. The sequence between the two minihomology sites is deleted.

The above data fit into a model in which the T4-encoded replication-recombination systems utilize minihomology sequences to join CRISPR-Cas-generated double-strand breaks. In the simplest model, the Cas9 genome editing complex introduces a double-strand break in the targeted protospacer sequence of the phage T4 genome (Fig. 8A). The 5′ ends of the cleaved DNA are then resected by an exonuclease generating 3′ overhangs of various lengths (Fig. 8B). The resected length might be shorter on the PAM-containing end because the CRISPR-Cas complex might remain bound to PAM for a length of time after cleavage (52). The 3′ overhangs anneal through minihomology sites and are stabilized by binding of UvsX and UvsY recombinase proteins to both single-stranded and annealed complexes, though having a higher affinity for single-stranded DNA (53, 54) (Fig. 8C). This is a critical step and may also explain why short stretches of homology are sufficient to generate paired complexes unlike the classic homologous recombination mechanism where quite long stretches of homology are required for efficient recombination. Pairing then leads to the assembly of replisome proteins, including gp43 DNA polymerase that initiate and extend DNA synthesis at the 3′ ends (55, 56) (Fig. 8D). Replication in both directions and digestion of overhangs by 5′ exonuclease would result in the stitching of Cas-cleaved ends. In the final step, nicks on both strands will be sealed by gp30 DNA ligase, generating an intact phage genome that is now completely resistant to CRISPR attack. Concatemerization by further cycles of DNA replication and processive packaging of “headful length” genomes into phage heads, each equivalent to ∼171 kbp, would lead to the assembly of phage particles. Since the phage T4 packaging machinery does not have strict sequence specificity as to where packaging begins or ends (57), these phages would carry a higher percentage of terminal redundancy than the parental phage, proportional to the length of the deletion. Unless the deletion is too long and resulted in a removal of an essential gene segment, these CRISPR-resistant mutant phages would survive and produce progeny plaques.

## DISCUSSION

Our studies uncovered a minihomology recombination mechanism in phage T4 that restores severed phage genomes generated by CRISPR-Cas cleavages. What is remarkable about this mechanism is that it pairs very short stretches of sequence identity, as few as 3 to 4 nucleotides, creating a novel substrate for DNA replication, repair, and covalent closure of broken ends. In comparison, the classic homologous recombination requires long stretches of sequence identity, at minimum ∼50 to 150 nucleotides in order to carry out a genetic exchange (26). The minihomology mechanism is also distinct from other CRISPR end-joining mechanisms reported in various host systems, including the most common nonhomologous end-joining (NHEJ) mechanism (58–64).

Our studies show that the success of the minihomology mechanism is predicated upon the presence of phage-encoded UvsX and other recombination, replication, and repair enzymes at the time of CRISPR cleavage. This means that the timing of CRISPR cleavage is critical. If cleavage occurred prior to the expression of T4 recombination/repair machinery, the broken genome will have little chance to survive because the ends would be rapidly degraded by the preexisting host nucleases, resulting in loss of essential genetic information and lethality (22). It is unknown precisely how long it takes for CRISPR-Cas complexes to scan the 10,584 Cas9 PAM sites or 13,776 Cas12a PAM sites of the invading phage T4 genome and target the protospacer sequence for cleavage. The available data indicate that it would be on the order of minutes (65), sufficient time for expression of early phage gene products following infection, which occurs within 1 to 5 min (66, 67). Therefore, it is likely that when CRISPR cleavage occurs, the T4 DNA replication, recombination, and repair proteins would have been expressed in a significant fraction of the infections. Hence, it is not entirely surprising that phage T4 evolved a robust mechanism by functional tuning of its DNA metabolism, such as acceptance by UvsX recombinase of short sequences for strand pairing and stabilization of paired complexes.

Another consequence of this functional tuning might be that the 3′ ends of the cleaved DNA can invade and pair with the complementary strands of the newly replicated T4 genome and initiate replication from the ends (see Fig. S5 in the supplemental material). This type of recombination-mediated strand invasion and replication has been well documented in T4, and in fact, it is a dominant mechanism for replication initiation since T4 genome does not contain a bona fide replication origin (68, 69). Eventually, these replicating complexes would resolve and the integrity of the CRISPR-cleaved genome would be restored except that, in this case, there would not be a deletion. Instead, as these replication/repair processes are error-prone, it would allow the selection of escape mutants at high frequency under CRISPR pressure. As reported here (Fig. 3 and 4) and previously (23), substitution mutations are also frequently identified in the escaped progeny phages. In fact, when the protospacer target is an essential gene, all the CRISPR escape mutants contained single-nucleotide substitutions, both missense and silent mutations (but no deletions), at the PAM and protospacer sequences. However, such single-nucleotide mutations have not been found in the minihomology-mediated deletion escape mutants, though these also require repair (Fig. 8), probably because deletion itself is sufficient for generating complete resistance to Cas cleavage and CRISPR escape. Thus, no additional pressure exists for selecting errors, unlike in the strand invasion mechanism where errors are the only means to generate Cas-resistant phage.

10.1128/mBio.01361-21.5 FIG S5 A model for UvsX-mediated homologous recombination of bacteriophage T4. (A) Cas nuclease cleaves the target DNA. (B) Resection of Cas-cleaved ends by exonucleases generating 3′ overhangs. (C) UvsY and/or UvsX bind to 3′-overhang single-stranded DNA and promote strand invasion of the intact genome. (D) Holliday junctions were generated by replication and ligation. Resolution of Holliday junctions restores genome integrity. Download FIG S5, TIF file, 1.7 MB. Copyright © 2021 Wu et al. 2021 Wu et al. https://creativecommons.org/licenses/by/4.0/ This content is distributed under the terms of the Creative Commons Attribution 4.0 International license.

The minihomology recombination mechanism confers broad selective advantages for phage survival. Long ago, Homyk and Weil (70) reported large deletions in the nonessential region between genes *39* and *56* (*39-56* region) (Fig. 2) when T4 mutants with duplications in the *rII* region were plated on an E. coli K(λ) lysogen that restricts *rII* mutants. In order to compensate for the duplication, phage must delete a nonessential region in order to produce a viable plaque because otherwise, the strictly headful packaging mechanism (57) will lead to loss of terminal redundancy due to the presence of additional duplicated region. When these mutants were sequenced by Mosig and colleagues (27), all the deletions contained repeats of short sequences flanking the deleted sequence (minihomology sites), as observed in the current study under CRISPR pressure. These were thought to be a result of “illegitimate” recombination, though these are likely due to legitimate UvsX-mediated minihomology recombination events triggered by double-strand breaks in the genome. Such breaks are expected to be extremely rare under normal conditions, consistent with the observed frequency of *39-56* deletion mutants on the order of 10^−8^ (70). It is tempting to consider that exposure to CRISPR-Cas systems during T4 evolutionary history provided unique opportunities for minihomology genetic exchanges and led to the selection of such escape mechanisms.

Finally, the importance of phage T4 genome modifications by glucosylation and cytosine hydroxymethylation (ghmC) in CRISPR escape should not be ignored. In fact, it is a critical player of phage’s multipronged counterdefense. It has been well documented that ghmC modification confers resistance against CRISPR-Cas cleavages and near complete blockage of most restriction enzyme activities (6, 7, 10). Consequently, phage gains a large repair and evolutionary space to stave off a variety of bacterial attacks. Resistance slows down CRISPR-Cas cleavages, which in turn creates a critical time window for phage genome to trigger a variety of responses: transcription and translation to synthesize early gene products, genome replication, recombination, and repair, anti-CRISPR molecules, and selection for mutants and escape mechanisms. Thus, genes such as the *uvsX* recombinase and modifications such as ghmC, which are considered “nonessential” under laboratory conditions, are indeed essential for phage survival in natural environment.

In conclusion, while bacteria evolved highly specific and potent mechanisms to destroy phage invaders, phages evolved broadly effective counterdefense and escape mechanisms by functional tuning of their DNA metabolism and selection of variants with far-reaching survival advantages. Thus, ironically, the very mechanisms that are designed to destroy bacteriophages apparently emerged as drivers for their greatest abundance and diversity on Earth.

## MATERIALS AND METHODS

### Plasmids.

CRISPR-LbCas12a/Cas9 plasmids were constructed using the streptomycin-resistant plasmid DS-SPCas as the starting plasmid (Addgene no. 48645). Sequences of spacers (listed in Table S1 in the supplemental material) were cloned into plasmid DS-SPCas in E. coli DH5α by overlap extension PCR (Thermo Fisher Phusion High-Fidelity PCR Master Mix) as previously described (9). Transformants were selected on streptomycin plates (50 μg/ml). The spacer-containing CRISPR-Cas9/Cas12a plasmids were extracted from the transformants, and the insertion of spacer sequences was confirmed by sequencing (Retrogen).

10.1128/mBio.01361-21.6 TABLE S1 Spacer sequences. Download Table S1, DOCX file, 0.01 MB. Copyright © 2021 Wu et al. 2021 Wu et al. https://creativecommons.org/licenses/by/4.0/ This content is distributed under the terms of the Creative Commons Attribution 4.0 International license.

The pET28b vector was used for construction of homologous donor plasmids to generate *uvsX.del* and *uvsY.del* mutant phages. For *uvsX.del* donor plasmid, two rounds of PCR were performed as previously described (9, 71). In the first round, the two homologous arms were amplified with primers listed in Table S2. In the second round, the two fragments were stitched to each other by including a 23-bp complementary region, where the *uvsX* deletion (Q52-G211) was introduced into, to form a full-length donor DNA. The donor DNA was then ligated into pET28b vector at the BglII and XhoI enzyme sites. A similar strategy was used to construct the *uvsY.del* (D5-F133) donor plasmid using primers shown in Table S2.

10.1128/mBio.01361-21.7 TABLE S2 Primers used for the genetic signature analysis of CRISPR escape plaques. Download Table S2, DOCX file, 0.01 MB. Copyright © 2021 Wu et al. 2021 Wu et al. https://creativecommons.org/licenses/by/4.0/ This content is distributed under the terms of the Creative Commons Attribution 4.0 International license.

### Bacteria and bacteriophages.

E. coli strains B834 (*hsdR*_B_ *hsdM*_B_ *met thi sup*^0^ *recA^+^*) was used for propagation of wild-type (WT) T4 phage, *uvsX.del*, and *uvsY.del* mutant phages and as plating bacteria to test the plating efficiency of various spacers (efficiency of plating of WT T4 phage [EOP_WT T4_]). E. coli DH5α [*hsdR17*(r_K_^–^ m_K_^+^) *sup*^2^ *recA*] was used for plasmid construction and spacer plating efficiency testing.

The WT T4 phage was prepared from our laboratory stock. The *uvsX.del* and *uvsY.del* mutant phages were constructed by CRISPR-Cas9 strategy in WT T4 phage background as described previously (9, 72). The spacer-containing CRISPR-Cas9 plasmid and the corresponding homologous donor plasmid were cotransformed into E. coli B834. E. coli cells either transformed with the donor plasmid or with the spacer-containing CRISPR-Cas9 plasmid were used as controls. E. coli cells containing spacer and donor plasmids and control E. coli cells were infected with WT T4 phage and the first generation (G1) recombined plaques were picked in 200 μl Pi-Mg buffer (26 mM Na_2_HPO_4_, 68 mM NaCl, 22 mM KH_2_PO_4_, 1 mM MgSO_4_ [pH 7.5]). Each plaque was purified under CRISPR pressure (G2). A single plaque from the G2 plate was picked into 200 μl Pi-Mg buffer to make the zero stock, and the mutated region was amplified by PCR and sequenced.

### Plaque assays.

The plaque assay was performed to determine the efficiency of spacer-expressing CRISPR E. coli to restrict T4 phage infection. As described previously (9, 23), serial dilutions of WT T4 phage (∼10^3^ to 10^6^ PFU) were added to E. coli (∼10^8^ cells/ml). The mixture (300 μl) was incubated for 7 min at 37°C, and then 3 ml of 0.7% top agar with streptomycin (50 μg/ml) was added, and the mixture was poured onto LB plate. After incubation at 37°C overnight, the first generation (G1) plaques were counted. The EOP (efficiency of plating) refers to the value determined by dividing the number of plaques produced by WT phage infection of CRISPR E. coli by the number of plaques produced by infection of control E. coli lacking the CRISPR plasmid.

### Evolutionary signatures of CRISPR escape plaques.

The evolutionary signature of each plaque was examined by PCR and DNA sequencing. Briefly, individual G1 CRISPR escape plaques were picked and put into 200 μl of Pi-Mg buffer, and 0.5 μl of each was used as a template for PCR amplification with a pair of primers flanking the protospacer target site (Table S2). The amplified DNAs were electrophoresed on an agarose gel, and individual bands from the agarose gel were sliced, the DNAs were extracted using QIAquick Gel Extraction kit (Qiagen) and sequenced (Retrogene). The sequences were then aligned with the WT sequence by BioEdit software to determine the mutation(s) introduced into phage genome.

### Minihomology sequence analyses.

Minihomology sequences flanking the CRISPR-cas9 cleavage sites were detected using the computing engine FAIR (Finding All Internal Repeats) (http://bioserver1.physics.iisc.ernet.in/fair/). The input sequences were stretches of genomic DNA covering the range of the longest deleted region for each spacer. All internal repeats of 3 or more nucleotides found within the input sequence were considered potential minihomology sites. The minihomologies were then sorted out by length of the sequence, GC content, distance to the PAM site, and frequency of usage, by SnapGene and Photoshop software programs.
