# Supplementary figures and images for: Bacteriophage T4 Escapes CRISPR Attack by Minihomology Recombination and Repair
Source: mBio. 2021 Jun 22;12(3):e01361-21. doi: 10.1128/mBio.01361-21 (PMC8262927; doi:10.1128/mBio.01361-21)

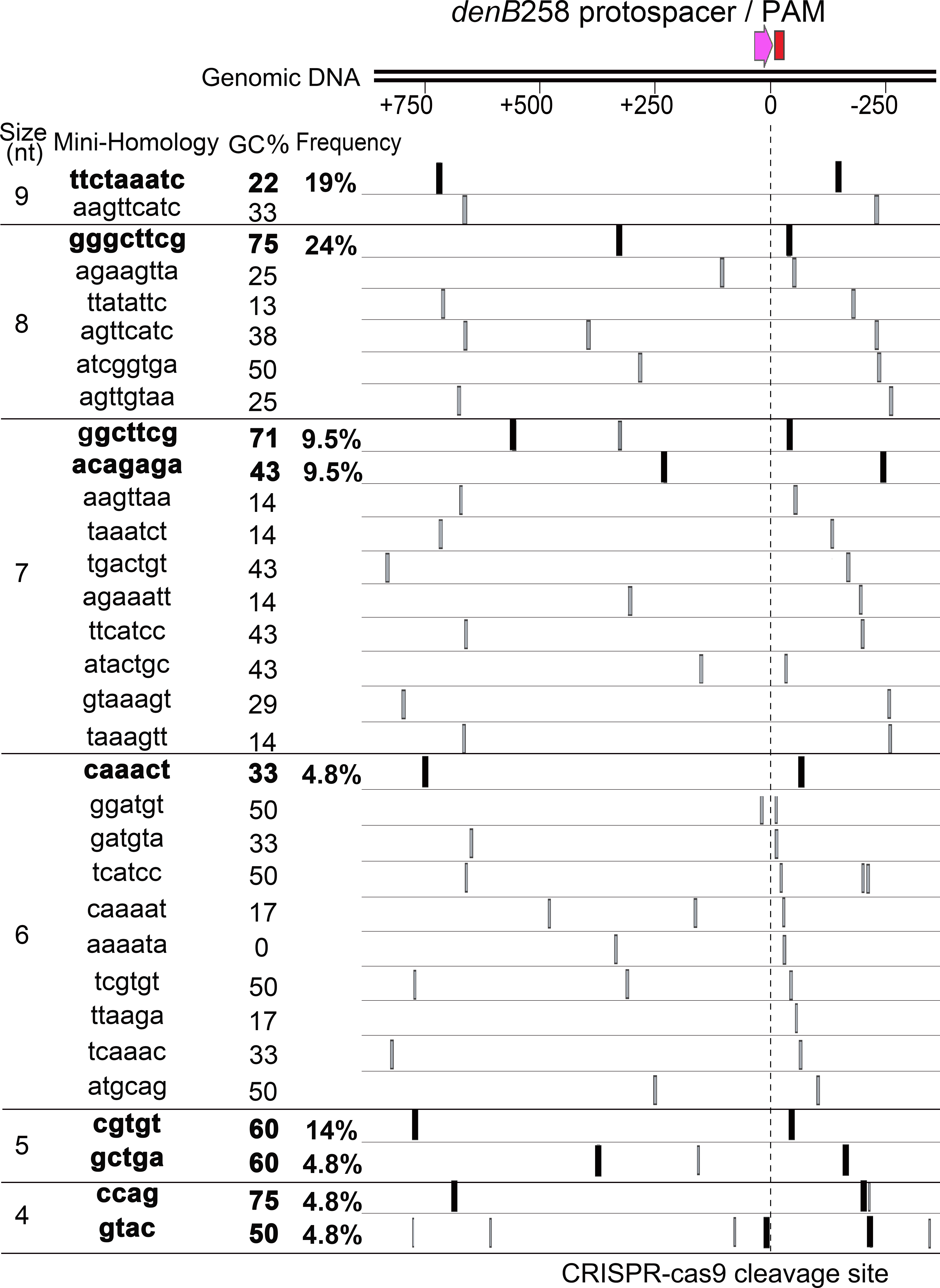

Supplement: FIG S1 [file mbio.01361-21-sf001.tif]

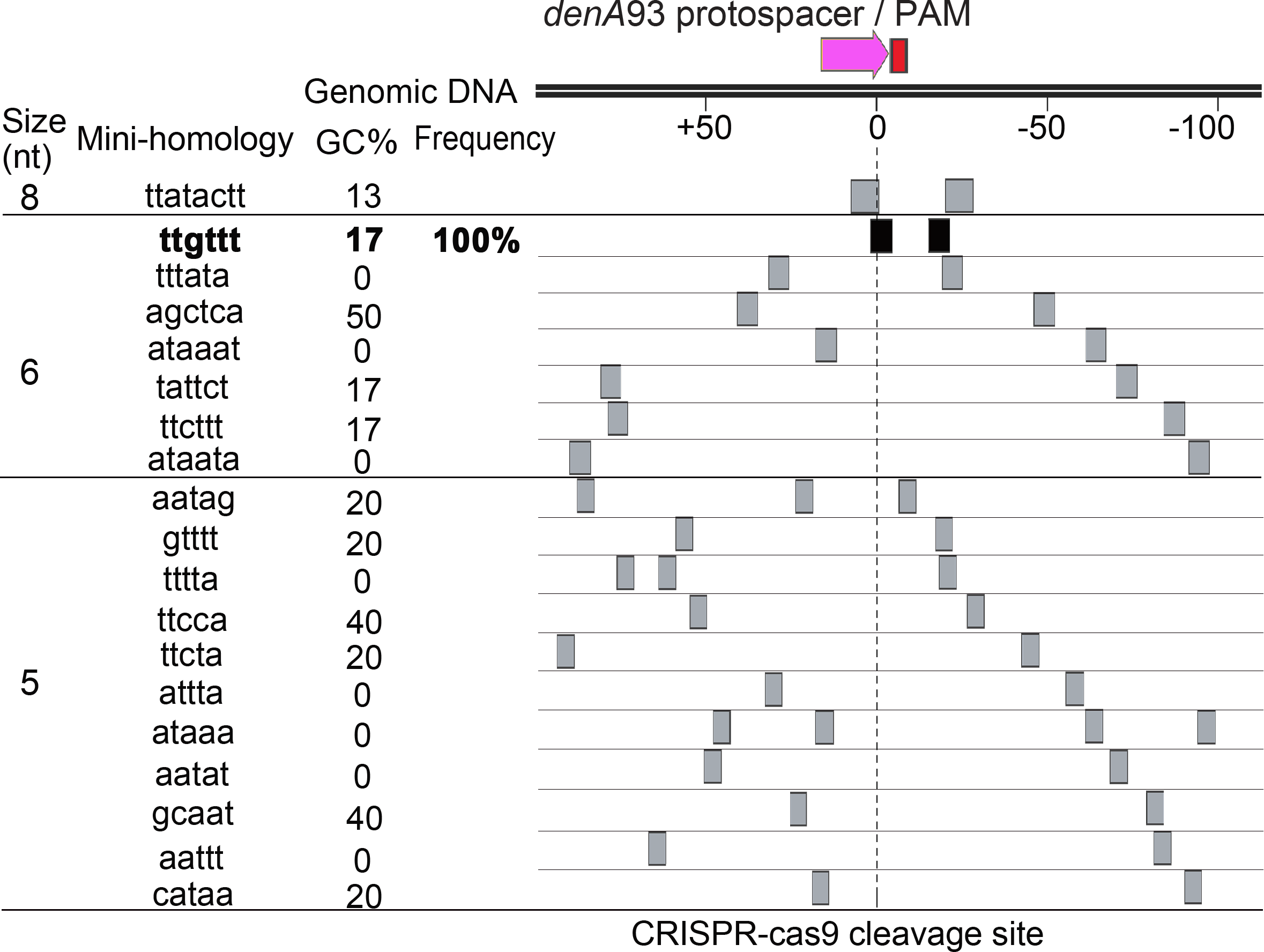

Supplement: FIG S2 [file mbio.01361-21-sf002.tif]

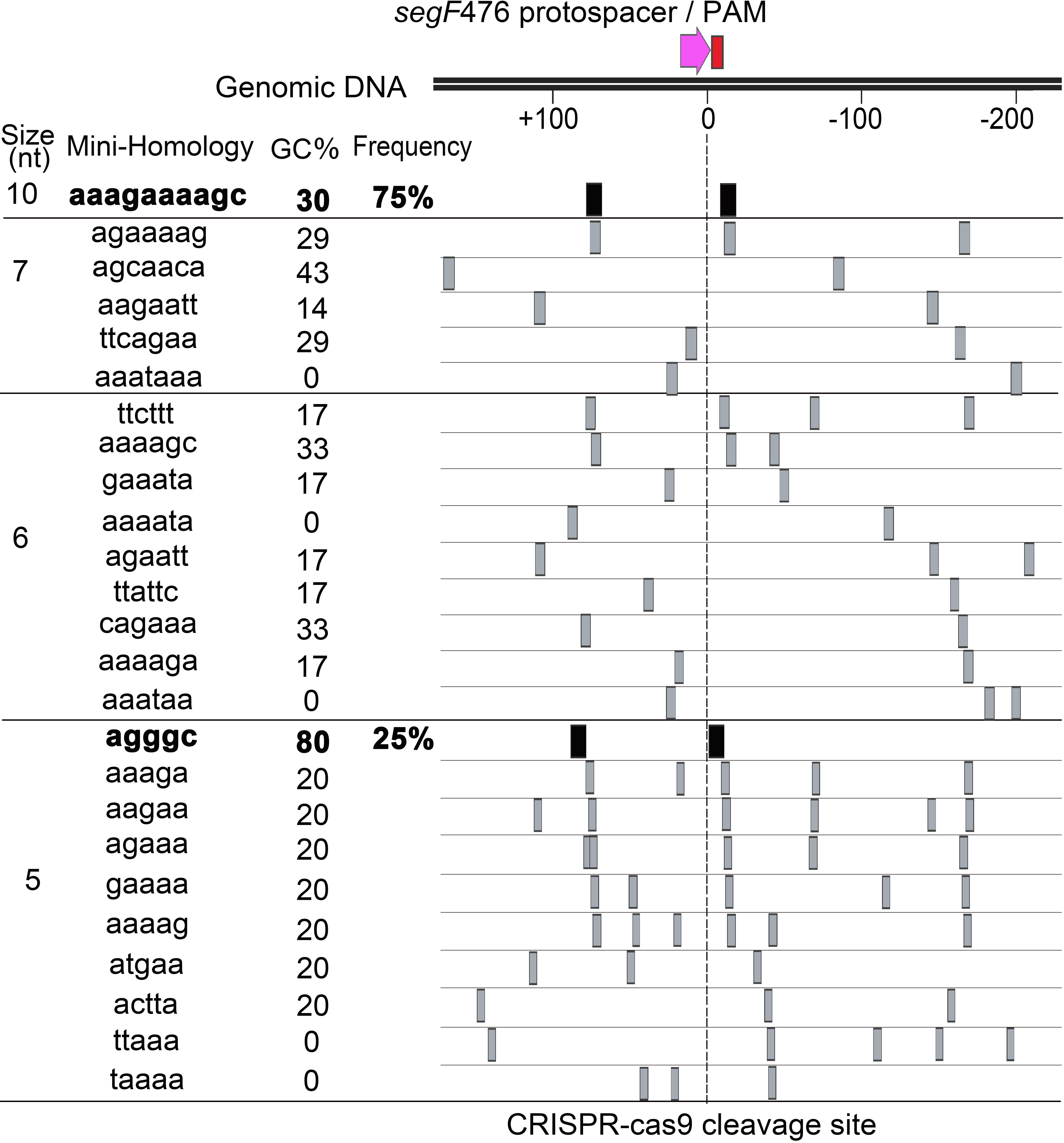

Supplement: FIG S3 [file mbio.01361-21-sf003.tif]

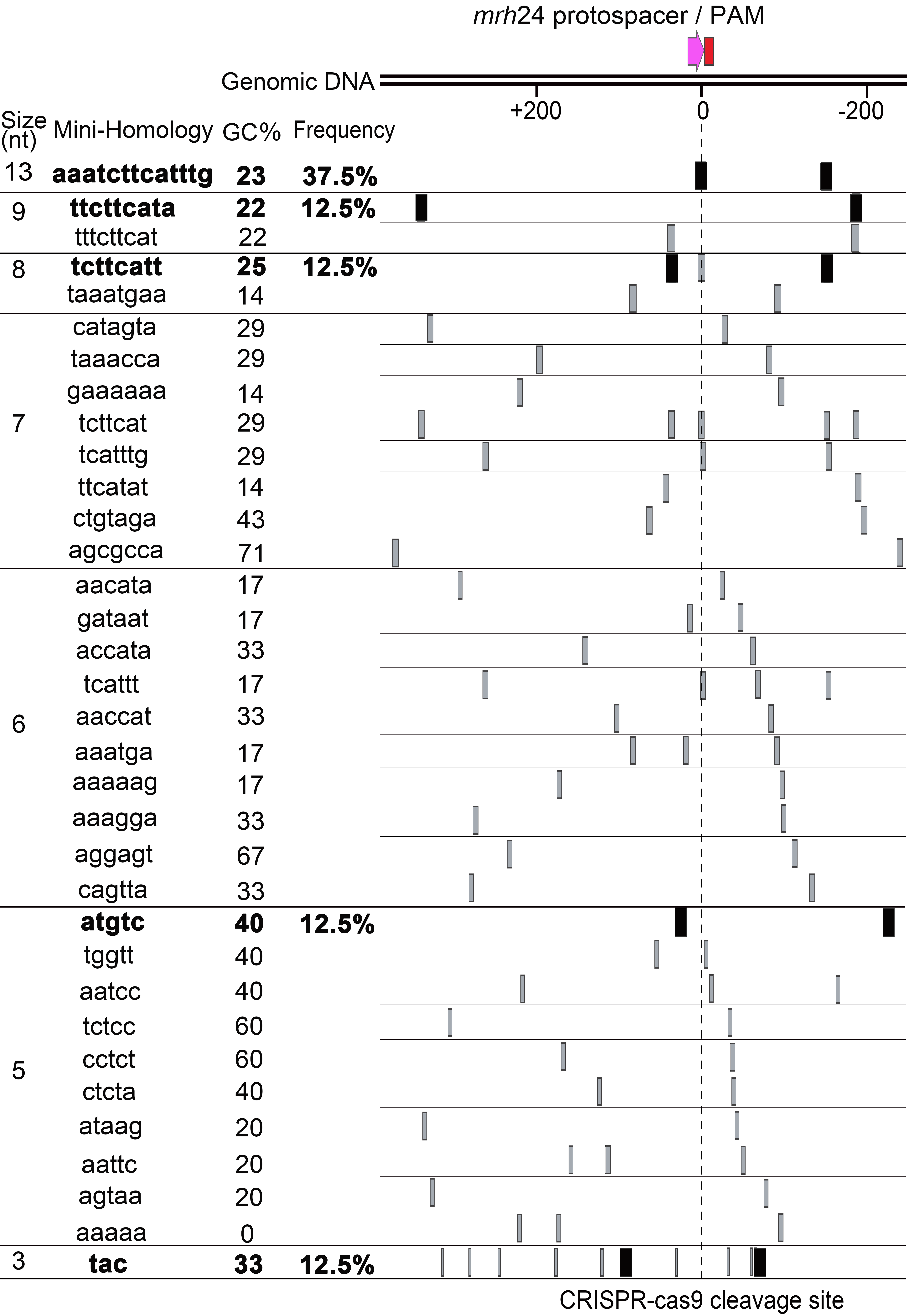

Supplement: FIG S4 [file mbio.01361-21-sf004.tif]

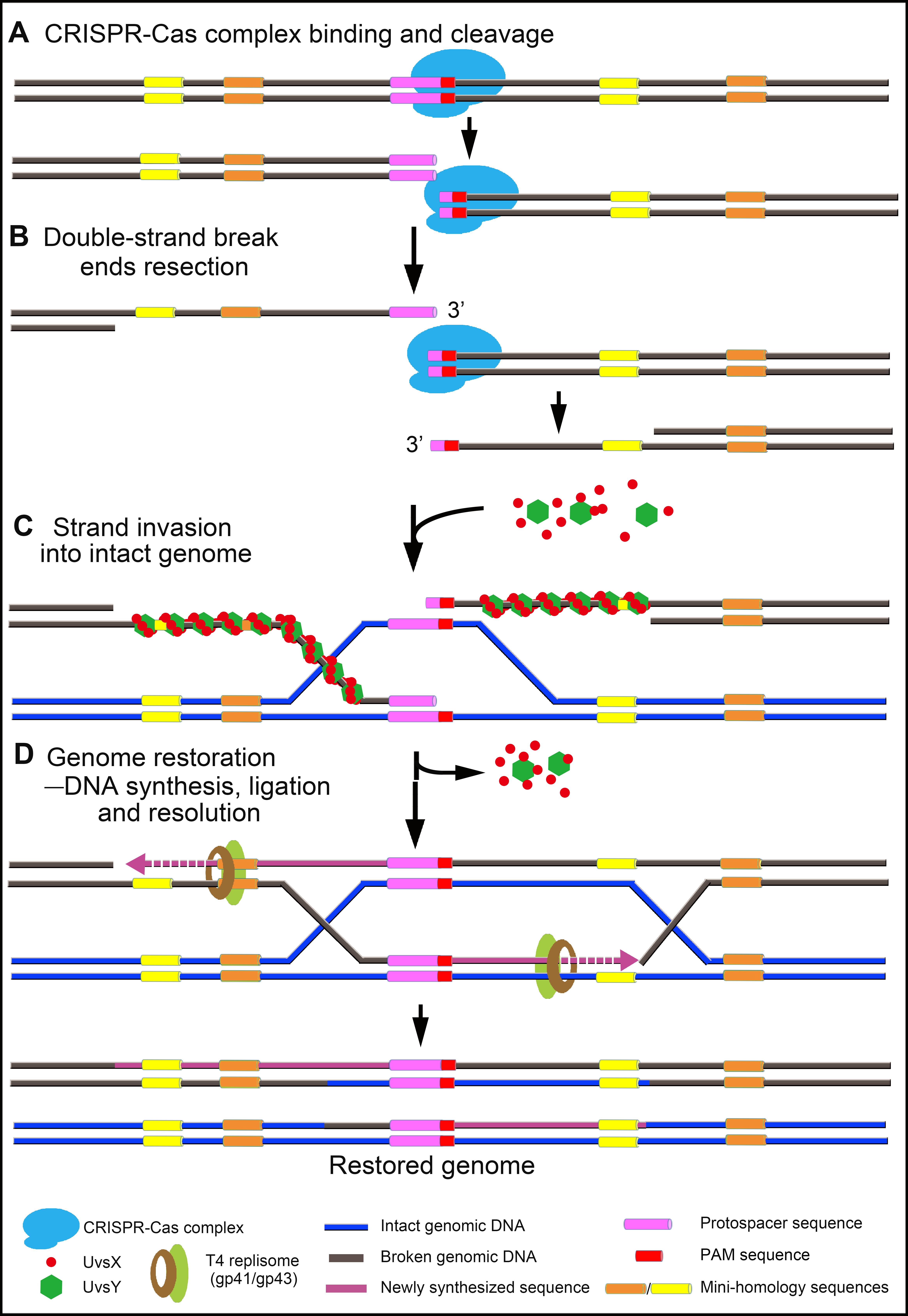

Supplement: FIG S5 [file mbio.01361-21-sf005.tif]
