# Supplementary material for: Bacteriophage T4 Escapes CRISPR Attack by Minihomology Recombination and Repair
Source: mBio. 2021 Jun 22;12(3):e01361-21. doi: 10.1128/mBio.01361-21 (PMC8262927; doi:10.1128/mBio.01361-21)
Supplement: TABLE S1 [file mbio.01361-21-st001.docx]

**TABLE S1** Spacer sequences

| **Protospacer name** | **Target gene** | **Sequence** |
| --- | --- | --- |
| *denA*93 | *denA* | 5’-taaattacgttatacttgtt-3’ |
| *denB*258 | *denB* | 5’-atgatccgtacacatatgca-3’ |
| *segF*476 | *segF* | 5’-gatttcagaaggaacttcaa-3’ |
| *mrh*24 | *mrh.2* | 5’-ataatatctaaatcttcatt-3’ |
| *uvsX*587 | *uvsX* | 5’-tttcttgtgtttcgtatgta-3’ |
| *uvsY*287 | *uvsY* | 5’-tgatacctcgttgcagtatt-3’ |
| *Cas12a-IPIII* | *IPIII* | 5’-cgtaaagaatttgccgatgt-3’ |
|  |  | 5’-ccgatgttatgactaaaggc-3’ |
| *Cas12a-far* | *far* region | 5’-tatcggttcaggtgaatcatcaa-3’ |
|  |  | 5’-acgggcatgcaaaccatagaaaa-3’ |
