# Supplementary material for: Bacteriophage T4 Escapes CRISPR Attack by Minihomology Recombination and Repair
Source: mBio. 2021 Jun 22;12(3):e01361-21. doi: 10.1128/mBio.01361-21 (PMC8262927; doi:10.1128/mBio.01361-21)
Supplement: TABLE S2 [file mbio.01361-21-st002.docx]

**TABLE S2** Primers used for the genetic signature analysis of CRISPR escape plaques

| **Gene** | **Primer sequences** | **Product size**  **size Size** | |
| --- | --- | --- | --- |
| *denA* | FW 5’-gttcttgcattgtgtatcctc-3’ | 1177 bp | |
|  | BW 5’-tggcacggcgacaattgca-3’ |  |  |
| *denB* | FW 5’-gttgtatagtaccacggtcc-3’ | 1286 bp | |
|  | BW 5’-agcggtatctcgactctttg-3’ |  | |
| *segF* | FW 5’-aaccgcgagtactagccatg-3’ | 410 bp | |
|  | BW 5’-ggactgagcaacaacgattag-3’ |  |  |
| *mrh.2* | FW 5’-tccacatgaatcacctgccca-3’ | 1061 bp | |
|  | BW 5’-caagggcgcaaagaaaagcac-3’ |  |  |
| *uvsX* | USFW (*Bgl*II) 5’-agAGATCTctcctggataacattttccat-3’ | 676 bp | |
|  | *del* USBW 5’-ctggtggtatgcctggaccgatgtattcggct-3’ |  |  |
|  | *del* DSFW 5’-catcggtccaggcataccaccagtaatttcac-3’ | 429 bp | |
|  | DSBW (*Xho*I) 5’-ttaCTCGAGcagctaggcaaaaagcagtc-3’ |  |  |
| *uvsY* | USFW (*Bgl*II) 5’-agAGATCTgggccaaaactatagactaaag-3’ | 461 bp | |
|  | *del* USBW 5’-gagattagaaggaggctggaaaataatgag-3’ |  |  |
|  | *del* DSFW 5’-ttccagcctcacttctaatctcatattgttc-3’ | 438 bp | |
|  | DSBW (*Xho*I) 5’-ttaCTCGAGggattggaacaaagacgtttc-3’ |  |  |
|  |  |  |  |
